# Oxygen Aspects in the High-Pressure and High-Temperature Sintering of Semiconductor Kesterite Cu_2_ZnSnS_4_ Nanopowders Prepared by a Mechanochemically-Assisted Synthesis Method

**DOI:** 10.3390/ijms24043159

**Published:** 2023-02-05

**Authors:** Katarzyna Lejda, Jerzy F. Janik, Marcin Perzanowski, Svitlana Stelmakh, Bogdan Pałosz

**Affiliations:** 1 Faculty of Energy and Fuels, AGH University of Science and Technology, al. Mickiewicza 30, 30-059 Kraków, Poland; 2 Institute of Nuclear Physics, Polish Academy of Sciences, ul. Radzikowskiego 152, 31-342 Kraków, Poland; 3 Institute of High Pressure Physics, Polish Academy of Sciences, ul. Sokołowska 29/37, 01-142 Warszawa, Poland

**Keywords:** kesterite Cu_2_ZnSnS_4_, semiconductor for photovoltaics, mechanochemical synthesis, nanopowders, oxygen contamination, high-pressure and high-temperature sintering, nanoceramics

## Abstract

We explore the important aspects of adventitious oxygen presence in nanopowders, as well as in the high-pressure and high-temperature-sintered nanoceramics of semiconductor kesterite Cu_2_ZnSnS_4_. The initial nanopowders were prepared via the mechanochemical synthesis route from two precursor systems, i.e., (i) a mixture of the constituent elements (Cu, Zn, Sn, and S), (ii) a mixture of the respective metal sulfides (Cu_2_S, ZnS, and SnS), and sulfur (S). They were made in each system in the form of both the raw powder of non-semiconducting cubic zincblende-type prekesterite and, after thermal treatment at 500 °C, of semiconductor tetragonal kesterite. Upon characterization, the nanopowders were subjected to high-pressure (7.7 GPa) and high-temperature (500 °C) sintering that afforded mechanically stable black pellets. Both the nanopowders and pellets were extensively characterized, employing such determinations as powder XRD, UV-Vis/FT-IR/Raman spectroscopies, solid-state ^65^Cu/^119^Sn NMR, TGA/DTA/MS, directly analyzed oxygen (O) and hydrogen (H) contents, BET specific surface area, helium density, and Vicker’s hardness (when applicable). The major findings are the unexpectedly high oxygen contents in the starting nanopowders, which are further revealed in the sintered pellets as crystalline SnO_2_. Additionally, the pressure–temperature–time conditions of the HP-HT sintering of the nanopowders are shown (in the relevant cases) to result in the conversion of the tetragonal kesterite into cubic zincblende polytype upon decompression.

## 1. Introduction

The quaternary sulfide Cu_2_ZnSnS_4_ (kesterite), or any member of the related substitutional derivatives (e.g., Se for S or Ge for Sn), is an outstanding direct semiconductor considered for the next generation of affordable and environmentally friendly photovoltaic (PV) cells to replace (for well-grounded reasons) widely utilized Si (various forms of silicon), CdTe (cadmium telluride), halide perovskites, CIGS (copper indium gallium diselenide), and a few other contenders [1,2,3,4]. Kesterite’s bandgap (tuned in the range 1.4–1.6 eV) and high light absorption coefficient (ca. 10^4^ cm^−1^) make it very well fitted for the absorber materials in thin-film solar cells. It is also stressed that the relatively high abundance of the elements forming kesterite and the environmental aspects of their utilization add to the fundamental advantages of the material. Specifically, kesterite nanopowders can be used as the inorganic part of the nanocontacts of an inorganic–organic PV cell [5] or as a sputtering source [6]. In this regard, the mechanochemical synthesis of kesterite nanopowders via the high-energy ball milling of its precursors appears to offer both simplicity and high potential for scaling up [7,8,9].

Kesterite is a non-oxide semiconductor, and its electronic properties will depend on nonintentional O substitutions in the preparation stage, whereas its long-term stability and functionality in device applications will rely on the material’s susceptibility to oxidation in humid air. This can be acutely true for those nanopowders with high specific surface area and an increased propensity for adsorption and chemisorption of gases such as oxygen and water vapor. Prolonged exposure to air could likely be associated with the progression of moisture-assisted oxidation and the essential uncertainty as to the extent of the reactions, possibly, from surface passivation to bulk consumption. These crucial O aspects are mostly unrecognized in studies on kesterite, with only a few nonsystematic reports on the subject. Theoretical calculations for pure and partially O-for-S-substituted kesterite (including the selenized form) in the series from Cu_2_ZnSnS_4_ via Cu_2_ZnSnS_x_O_4−x_ (0 > x > 4) to Cu_2_ZnSnO_4_ supported the exothermic reactions of kesterite with oxygen and, hence, such oxidation is very likely to occur [10]. The calculations also provided hints as to the changes in the crystallographic cell parameters and to the increase of both the energy bandgap and absorption efficiency upon oxygen substitution [10,11]. The influence of intentional and rather high O substitutions in kesterite films in prototypical PV cells on their optoelectronic properties [12], as well as of postsynthesis residual oxygen or annealing in air on cell stability and efficiency [13,14], were reported. Interestingly and symptomatically, both the positive and negative effects of PV cell annealing in air were occasionally noted, which were ascribed to the passivation of the grain/film interface boundaries [15]. There were also rare studies on the characterization of kesterite films or powders, which reported the detection of some O-bearing compounds, metal oxides and/or Cu and Zn-sulfates upon synthesis and storage under ambient conditions [16,17,18], as well as the intentional heating in air [19], mostly by applying X-ray photoelectron spectroscopy (XPS) and Raman spectroscopy. Our previous study on the magnetic aspects of kesterite nanopowder synthesis, which was linked to kesterite partial oxidation/lattice disorder and the presence of Cu^+2^ ions, revealed the detrimental impact of magnetism on the material’s semiconducting properties [20]. In summary, oxygen seems unavoidable and, sometimes, intentional in kesterite materials, and if not purposefully analyzed, it will then incidentally reveals its presence.

One of the research targets in nanopowder utilization is its conversion to a more manageable material form, including, for instance, solid layers or compacts made by various methods. With regard to kesterite nanopowders, the term “sintering” has been occasionally and rather misleadingly applied to describe a final stage in a multi-step process starting with either the thin-film deposition of precursors [3,21,22] or the cold-pressing of raw powders [23], followed by thermal annealing at sufficiently high temperatures. Such processing results, first, in solid layers or compacts that are later subjected to temperature-promoted reactions/changes, yielding the target tetragonal kesterite. However, such an end-product can hardly be described as having been sintered in a widely accepted meaning of the term, i.e., formed in a temperature/pressure-induced process involving the atoms in the materials, which diffuse across the particle surfaces and make binding sites, connecting them together into one piece without the necessity of chemical reactions taking place [24]. There are also rare reports referring to the hot-pressing of kesterite powders to be served as sputtering targets, but, for instance, the applied pressures are relatively low (in the order of a few tens of MPa) [25] when compared with the available pressures of several GPa accompanied by temperatures of up to 1000 °C and higher, as is potentially applied in standard high-pressure (HP) and high-temperature (HT) sintering [24].

Herein, a comprehensive set of kesterite nanopowders is prepared via the high energy ball milling from two different precursor systems, as previously reported in detail by us, i.e., from the mixture of the constituent elements {2Cu + Zn + Sn + 4S ➔ Cu_2_ZnSnS_4_} [8,20] and from the mixture of metal sulfides and sulfur {Cu_2_S + ZnS + SnS + S ➔ Cu_2_ZnSnS_4_} [20]. In each system, the raw powder is isolated after milling and upon the removal of the solvent. This is followed by thermal annealing at 500 °C under argon, which yields the final material. The specific mechanochemical synthesis conditions result in the isolated raw powders being made of the kesterite’s cubic polytype with defunct semiconductor properties, which is tentatively called by us prekesterite. The subsequent treatment of the prekesterite nanopowder at 500 °C is essential to convert it to the thermodynamically stable tetragonal kesterite with semiconductor properties. The prekesterite and kesterite nanopowders from both synthesis routes are subjected to high-pressure (7.7 GPa) and high-temperature (500 °C) sintering to afford mechanically robust nanoceramics that are extensively characterized. In this regard, the application of temperatures exceeding 550–600 °C is avoided due to the thermal instability of the kesterite nanopowders. The HP-HT sintering methodology applied to kesterite was mastered by us during recent studies on the sintering of nanopowders of the metal nitrides, including AlN, GaN, TiN, and their composites [26,27,28]. 

## 2. Results and Discussion

### 2.1. Powder Materials Stage

**Figure 1:**
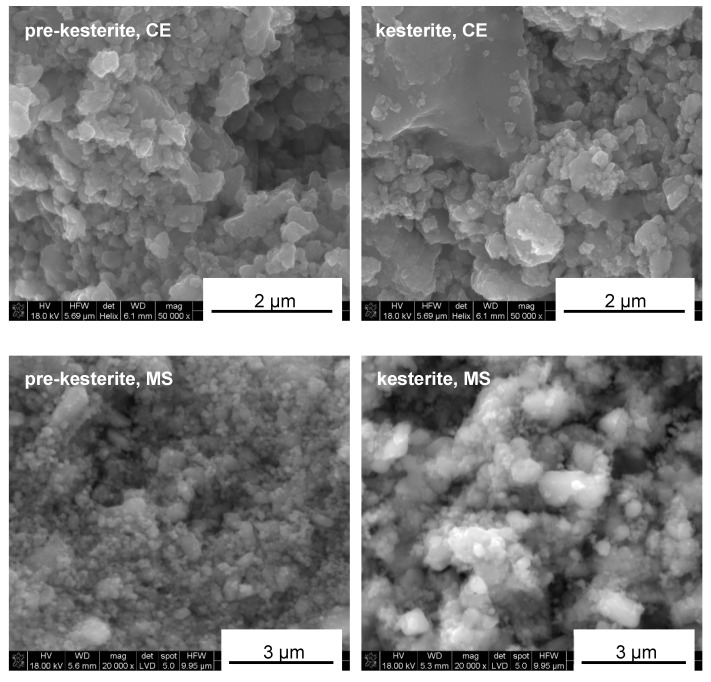
Typical nanopowder morphology, as examined by SEM for products in the CE system (**top** row) and MS system (**bottom** row).

The prekesterite (raw after milling) and kesterite (heat-treated at 500 °C) materials made from the two precursor systems are blackish powders. Their nano-sized features are evident from an examination of the respective scanning electron microscopy (SEM) images. For comparison, Figure 1 shows the morphologies typical for the prekesterite and kesterite from both systems. It is apparent that the morphologies are very similar in that they show irregularly shaped agglomerates/particles with very alike-size distributions and varying dimensions from a few micrometers down to below 50–100 nanometers. It is instructive to point out that the smallest discernible particles can still be agglomerates of even smaller crystallites in the low nano-sized regime, and this aspect will be further elucidated based on the relevant X-ray diffraction (XRD) patterns.

**Figure 2:**
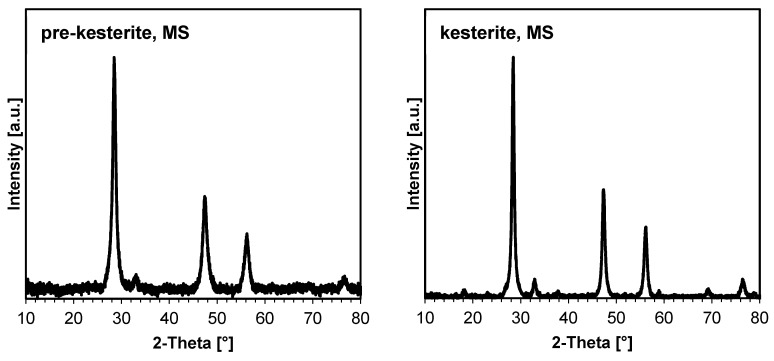
XRD patterns for nanopowders in the MS system: (**left)**—cubic zincblende-type prekesterite (F43m); (**right)**—disordered tetragonal kesterite (I42m).

The XRD patterns for the prekesterite and kesterite powders are typical for the compound and are identical for both systems, as already reported [8,20]. For illustration, Figure 2 shows the patterns for the MS case, whereas Table 1 contains the essential parameters calculated from the patterns for all powders in both systems. The data confirm the powders’ nanoscopic characteristics. The raw prekesterites prepared at ambient conditions have average crystallite sizes in the order of 8–10 nm. The two kesterite samples after 500 °C annealing are also similar to each other but with larger sizes of 14–16 nm, which is consistent with the accompanying recrystallization/crystal growth processes after heating, as reported earlier by us [8,20]. Such consistency adds to the advantages of the mechanochemical synthesis method using different precursor systems.

**Figure 3:**
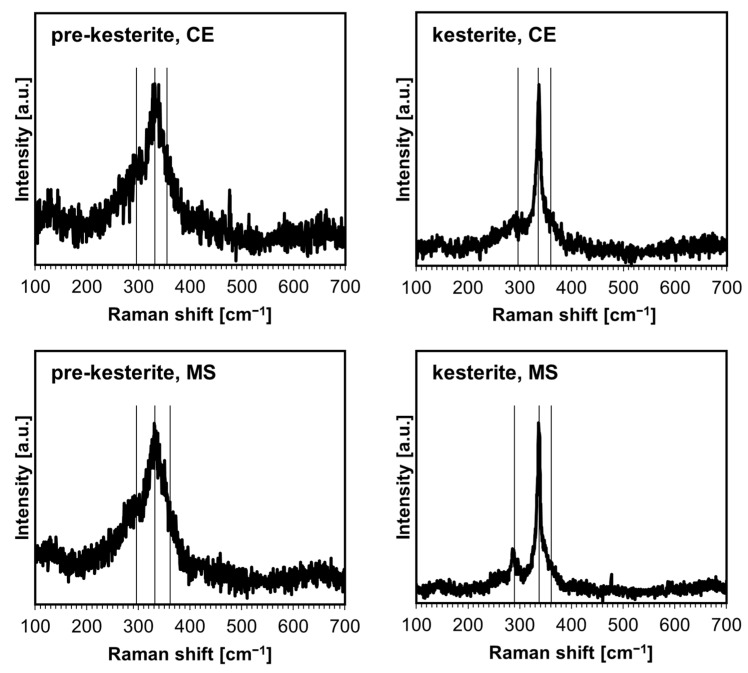
Raman spectra for the nanopowders from the CE system (**top** row) and MS system (**bottom** row). Vertical lines indicate the approximate positions of kesterite-specific Raman bands.

**Figure 4:**
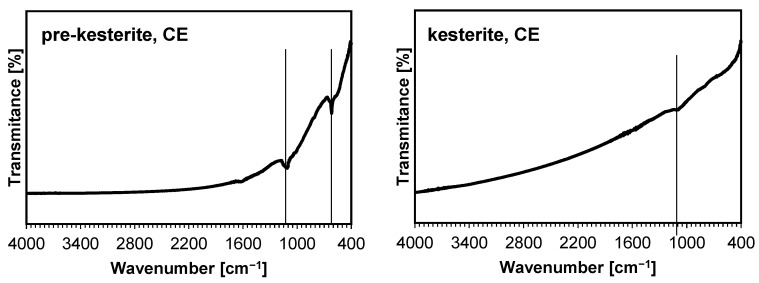
FT-IR spectra for nanopowders from the CE system. Vertical lines show the positions of the bands typical for various metal sulfates.

The characterization of the materials by Raman spectroscopy does not pose any side effects regarding sample overheating by laser beam and/or with fluorescence and provides data consistent with the experimental spectroscopic features for prekesterite and kesterite [8]. It has been reported that the A symmetry modes in the range 290–340 cm^−1^ are the strongest and, together with a rather weak B symmetry mode at 350–360 cm^−1^, are the signature bands for kesterite [29]. At the same time, these bands can be significantly broadened and overlapped for a low-quality polycrystalline kesterite, which refers to the nano-sized and defective material characteristics. The spectra for the powders from both systems are shown in Figure 3. The spectra for the prekesterites can be satisfactorily deconvoluted into three rather broad superimposed bands at 292–297 cm^−1^ (weak; v. broad), 332–336 cm^−1^ (strong; broad), and 350–360 cm^−1^ (v. weak; broad shoulder). The spectra for the kesterites have the respective bands of 290–295 cm^−1^ (weak; broad), 336–338 cm^−1^ (strong, sharp), and 355–360 cm^−1^ (v. weak; shoulder). In general, the spectra for both varieties are similar, while the corresponding bands for the kesterites are better shaped up, with the major and strongest band of A symmetry at 336–338 cm^−1^ being visibly sharper. This is in agreement with the related crystallographic and chemical nature of both polytypes and the better crystallinity of the annealed kesterite vs. raw prekesterite, as judged also by the larger average crystallite size of the former (Table 1). Finally, there are no clearly defined additional bands, especially in the range of ca. 1000–1200 cm^−1^, which could be convincingly linked to the sulfate −SO_4_ groups in plausible surface oxidation products. On the other hand, the Fourier transform infrared (FT-IR) spectra confirmed some products of this type, as is illustrated in an example for the CE system in Figure 4. Regarding this, kesterite does not have any specific absorption bands in the mid-infrared range. The band seen at ca. 1050–1200 cm^−1^ is found in such inorganic sulfates as CuSO_4_ and ZnSO_4_ and their hydrated derivatives [30,31]. The additional band present for the prekesterite powder (Figure 4, left) at 610 cm^−1^ is likely associated with the hydrated zinc sulfate ZnSO_4_·7H_2_O that shows two strong bands at ca. 1100 and 610 cm^−1^ [31]. In conclusion, the infrared spectra for the nanopowders support some low yet detectable amounts of the sulfate-type oxidation byproducts.

**Figure 5:**
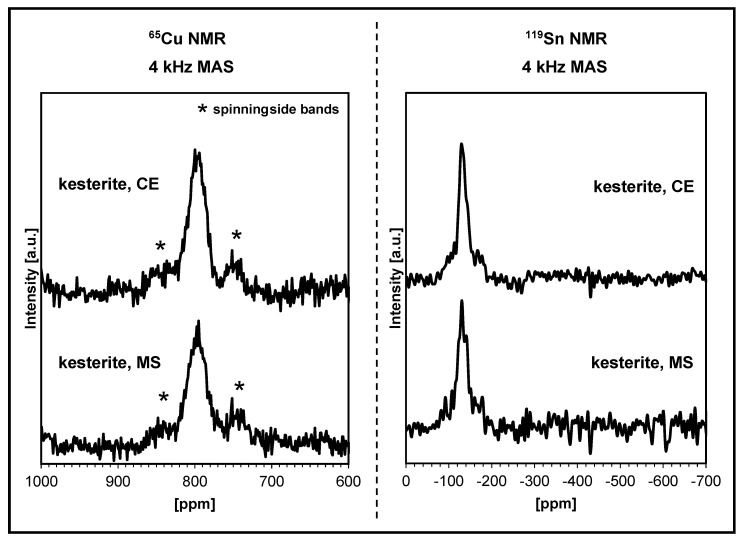
Solid-state MAS NMR spectra for kesterite nanopowders: (**left**)—^65^Cu NMR, (**right**)—^119^Sn NMR. In both sides, the upper spectra are for the CE system and the lower spectra are for the MS system.

The ^65^Cu and ^119^Sn solid-state magic angle spinning nuclear magnetic resonance (MAS NMR) measurements were attempted for both the prekesterite and kesterite powders. In agreement with the observations previously reported by us, the prekesterite powders do not produce any ^65^Cu or ^119^Sn MAS NMR spectra [8,20,32,33]. This is ascribed to the inherent d_0_ magnetism of the raw powders, which is linked directly to the paramagnetic Cu^+2^ centers originating from lattice disorder and the adventitious oxidation of some diamagnetic Cu^+1^ sites. On the other hand, both kesterite powders are active in such experiments, producing the anticipated spectra. Figure 5 shows the ^65^Cu and ^119^Sn NMR spectra for the kesterite nanopowders from both the precursor systems with resonances in the expected copper and tin chemical shift ranges [20,32,33]. In this regard, the single ^65^Cu resonance at ca. 795 ppm is characteristic of a quadrupolar-dominated central transition pattern, whereas the single and symmetrical ^119^Sn NMR peak at ca. −128 ppm reflects a relatively high degree of lattice ordering in the kesterite.

**Figure 6:**
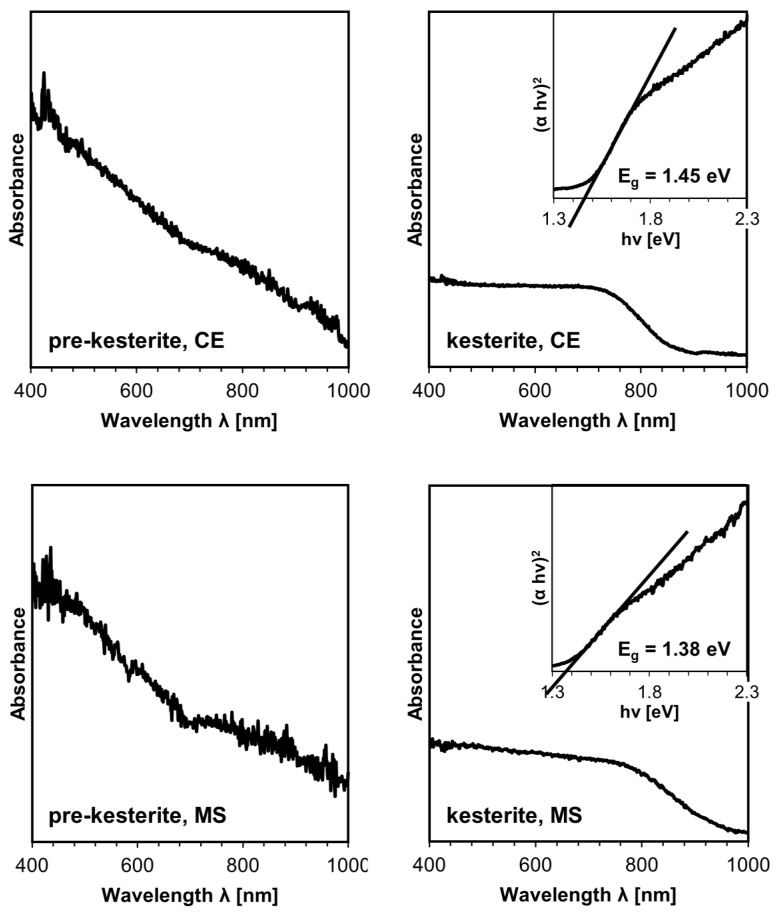
UV-vis spectra for the nanopowders from the CE system (**top** row) and MS system (**bottom** row). Spectra for the annealed kesterites have inserts of Tauc (αhν)^2^ vs. hν [energy] plots (α approximated by Kubelka–Munk transformation) and include the calculated energy band gaps E_g_. Note the nonspecific spectra for both prekesterites with defunct semiconductor properties.

The characteristic property of kesterite impacting its applications in PV solar cells is the energy band gap E_g_, which is conveniently determined by ultraviolet-visible (UV-vis) spectroscopy. Figure 6 shows the UV-vis spectra for the prekesterite and kesterite powders from the CE and MS systems. It is instructive to recall that the raw prekesterite has defunct semiconductor properties and does not show any specific energy band gap [8,20]. This is illustrated in the plots for the prekesterites that show nonspecific absorbance spectra. Both kesterite nanopowders have spectra that provide (via Tauc (αhν)^2^ vs. hν [energy] plots) slightly different values for the energy band gaps of 1.45 and 1.38 eV for the CE and MS systems, respectively. The small difference is thought to stem from the specifics of the mechanochemical synthesis of kesterite in the two precursor systems, resulting in compositionally disordered products with slightly different spectral properties. This disorder may further determine their varying susceptibility to surface oxidation at ambient conditions.

Regarding the issue of kesterite particle surface oxidation, it is worth noticing the relatively high and similar surface areas for all the nanopowders. In the CE system, the determined BET (Brunauer–Emmett–Teller) specific surface area is 18.2 m^2^/g for prekesterite and 17.3 m^2^/g for kesterite, whereas, in the MS system, these values for prekesterite and kesterite are 17.9 and 17.2 m^2^/g, respectively. These magnitudes for the specific surface area reflect the prevailing mesoporosity of the nanopowders, as supported by the derived values of the BJH (Barrett–Joyner–Halenda) mesopore area in the range 21–22 m^2^/g. It is also important to underline that, additionally, there is some closed porosity present, as supported by the helium density d_He_ measurements. The d_He_ values for cubic prekesterite and tetragonal kesterite in the CE system are 3.84 and 4.12 g/cm^3^, and, in the MS system, they are 3.90 and 4.24 g/cm^3^, respectively. These can be compared to the theoretical/calculated value of 4.56 g/cm^3^ for tetragonal kesterite (stannite structure) [34]. The now-determined densities are clearly lower, supporting the share of the closed pores inaccessible to helium penetration and also, possibly, the presence of distinct structural defects in the nanopowders [8].

**Figure 7:**
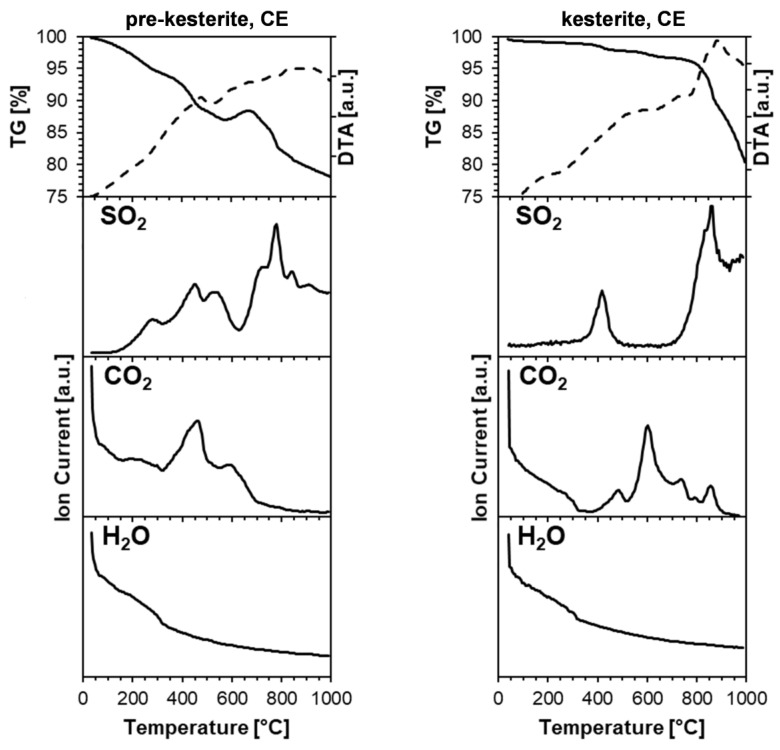
TGA-DTA/MS data up to 1000 °C for the nanopowders of prekesterite (**left** column) and kesterite (**right** column) from the CE system. In the top row, the solid-line curve depicts thermogravimetric TGA changes, and the dashed-line curve shows thermal DTA changes. In the rows below, the evolution of the selected gases containing oxygen on heating is displayed (consecutively, from top to bottom, SO_2_, CO_2_, and H_2_O).

The thermogravimetric analysis-differential thermal analysis (TGA-DTA) data coupled with mass spectroscopy (MS) of the evolving gas species provide strong evidence of some oxygen content in the nanopowders. In this regard, the measurements were carried out under high-purity argon so as to minimize possible carrier gas oxygen contamination interference. Figure 7 shows the data acquired for the prekesterite (left) and kesterite (right) nanopowders from CE system, which include the TG and DTA curves, as well as the traces of the evolution of oxygen-containing gases such as sulfur dioxide SO_2_ (SO_3_ is not detected), carbon dioxide CO_2_, and water vapor H_2_O.

For the prekesterite nanopowder (Figure 7, top left), there is a three-step ca. 8% sample mass loss up to 600 °C, the latter being a temperature stability range for such nanopowders. Past 660–670 °C and up to 1000 °C, a continuous mass decrease due to sample decomposition is observed down to the final 77% of the initial sample mass. The sulfur dioxide SO_2_ evolution curve shows three major evolution events up to 600 °C and a few more in the 700–900 °C range. All these events coincide with distinct mass loss steps, supporting the view that the evolution of SO_2_ is mostly responsible for them. The most likely source of SO_2_ is the decomposition of the metal sulfate salts, e.g., plausible ZnSO_4_, CuSO_4_, and their hydrates formed as oxidation byproducts on particle surfaces during sample handling and manipulations in ambient air [20]. For instance, an overall zinc sulfate decomposition reaction up to 1000 °C, which goes through transient SO_3_ formation, can be written as ZnSO_4_ ➔ ZnO + SO_2_ + ½O_2_ [35]. Almost complete CuSO_4_·5H_2_O decomposition, initially with dehydration steps at up to 250 °C, and then with formation of CuO and release of SO_2_ takes place in the 650–750 °C range [36]. An interesting observation is linked with the carbon dioxide CO_2_ evolution (Figure 7, left). First, at the beginning of heating below 80–100 °C, a steep decrease in gas evolution takes place, which is typical for desorbing CO_2_. Second, there is an increase in the evolution of CO_2_ at 350–600 °C (two peaks at 450 and 600 °C), which is subdued past 650 °C. This can rationally be explained as being caused by the oxidation of the residual carbon species in the sample by adventitious oxygen in the argon carrier gas. Another rationale for carbon oxidation to CO_2_ is carbon’s reaction with sulfur trioxide SO_3_ during the initial stages of sulfate salt decomposition, i.e., C + 2SO_3_ ➔ CO_2_ + 2SO_2_. This is consistent with the presence of the relatively low-temperature SO_2_ peaks, i.e., below 400–600 °C. In this regard, the carbon source can result from the pyrolytic remnants of the xylene that is used in the synthesis stage and, apparently, has not been completely removed during sample drying by evaporation. The fact that one observes this CO_2_ effect in all prekesterite/kesterite samples and in all thermogravimetric measurements supports such a supposition. Finally, the trace of H_2_O evolution (Figure 7, bottom left) is typical for a desorbed gas, while some relatively increased quantities of the component in the 100–300 °C range vs. the higher temperatures may be indicative of some contribution from metal sulfate dehydration.

The TGA/DTA determination for the kesterite nanopowder (Figure 7, top right) provides us with a multistep ca. 2% combined mass loss up to 700 °C, whereas, from 760 to 1000 °C, a pronounced mass loss starts due to compound decomposition, which continues down to ca. 80% of the initial mass. It is worth noting the much smaller mass loss and larger stability range of kesterite when compared to the related prekesterite (vide intra), which is consistent with the better crystallinity and stability of the former. There are two well-defined distinct SO_2_ evolution events—the first one peaking at 420 °C, and the second one at 840 °C; the latter is both within the kesterite decomposition range and the final steps of decomposition of ZnSO_4_ [35]. There are a few CO_2_ peaks above 400 °C, with the most intense at 600 °C, but with the less intense ones also at 730 and 850 °C. They likely reflect the reactions of SO_3_ with C, as outlined above. Finally, the H_2_O evolution curve is similar to the already discussed case of prekesterite and is consistent with water vapor desorption at lower temperatures and the possible dehydration of the sulfates at higher temperatures.

Unequivocal evidence for the oxygen content in the nanopowders is provided by direct O and H content determinations. Table 2 contains the relevant data acquired for all the powder products. It appears that the O contents are unexpectedly high (of the order of a few weight percent) both for the prekesterite and kesterite. Given the rather low oxygen contents in the starting materials in both precursor systems (of the order of a few tenths of a percent), this suggests the high propensity of the nanopowders to adventitious oxidation on a few occasions when exposed to air during the necessary sample handling and manipulations. This also suggests that special precautionary measures must be undertaken to minimize this exposure. The H contents may be used to estimate a part of the oxygen bound as H_2_O. For instance, the 0.37 wt% H content for prekesterite in the CE system supports ca. 3 wt% of O content involved in H_2_O out of the determined total of 5.06 wt%, while the relevant 0.12 wt% for prekesterite in the MS system is equivalent to ca. 1 wt% O content in H_2_O out of the total 3.85 wt%. Based on the FT-IR and TGA-DTA/MS data, the remaining part of the oxygen is mostly associated with the sulfate −SO_4_ groups as a result of the compound’s noticeable susceptibility to oxidation to metal sulfates in ambient air. The prekesterite/kesterite nanopowders are especially prone to such oxidation, owing to their increased surface area coupled with reactive defected structures. A follow-up study has been in progress to address the kesterite oxidation in air on a several-month time scale, including its impact on chemical integrity and crucial semiconductor properties. It will address the significance of substrate O purity and the oxygen footprint for kesterite from the synthesis stage (via material manipulations) to long storage.

### 2.2. Sintered Nanoceramics Stage

**Figure 8:**
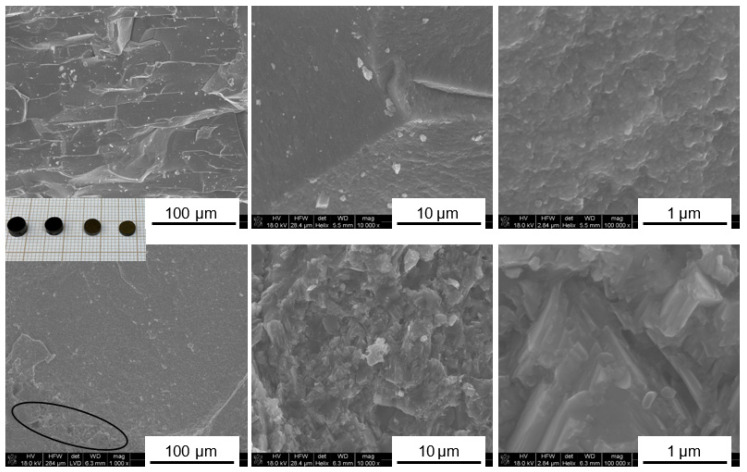
SEM images for the fractured pellets of prekesterite (**top** row) and kesterite (**bottom** row), which were prepared from the respective nanopowders in the CE system. Insert on the left—a snapshot of sintered pellets. Note a small region of macroporosity in kesterite shown in oval, in which micro-sized texturing is observed (lower row).

The morphological features of the pellets prepared by high-pressure and high-temperature sintering of the prekesterite and kesterite nanopowders in the CE system are exemplified in Figure 8 and are typical for nanoceramics in both systems. The fractured fragments show dense surfaces of uniform morphology. Under higher magnification (Figure 8; middle and right columns), the morphology of the prekesterite is grainy and quite homogeneous, whereas that of kesterite shows some texturing on the micrometer scale, especially for regions with apparent macropores. The magnification of such a region (as enclosed in an oval shape in Figure 8) illustrates this feature with some directionally preferential/layered structures. It is worth pointing out that there is some closed porosity present in all pellets, supported by the helium density measurements, that can be related to the reference density of 4.56 g/cm^3^ for kesterite [34]. In this regard, the helium density d_He_ in the CE system is determined for the prekesterite pellet at 3.88 g/cm^3^ and 4.28 g/cm^3^ for the kesterite pellet, whereas in the MS system, the respective densities are 3.93 g/cm^3^ and 4.30 g/cm^3^. It is also interesting to note that these densities are very similar to the densities of the parent nanopowders, as discussed earlier, i.e., for the prekesterite and kesterite in the CE system—3.84 and 4.12 g/cm^3^ and in the MS system—3.90 and 4.24 g/cm^3^, respectively, so, essentially, there is only a minute density increase from the high-pressure and high-temperature sintering.

The Vicker’s hardness (H_V_) data for the nanoceramics are moderately high and reflect the pellets’ mechanically robust nature. In the CE system, the determined H_V_ values for the sintered prekesterite and kesterite pellets are 2.5 and 3.1 GPa, and in the MS system, they are 1.5 and 2.7 GPa, respectively. These numbers can be related to some scarce literature data for natural kesterite minerals or the available theoretical approximations that are all in the range 2.2–3.9 GPa [37,38]. The H_V_ values are higher for the sintered kesterite nanopowders compared with the sintered prekesterite nanopowders, matching the trend in helium densities for the respective pellets. 

**Figure 9:**
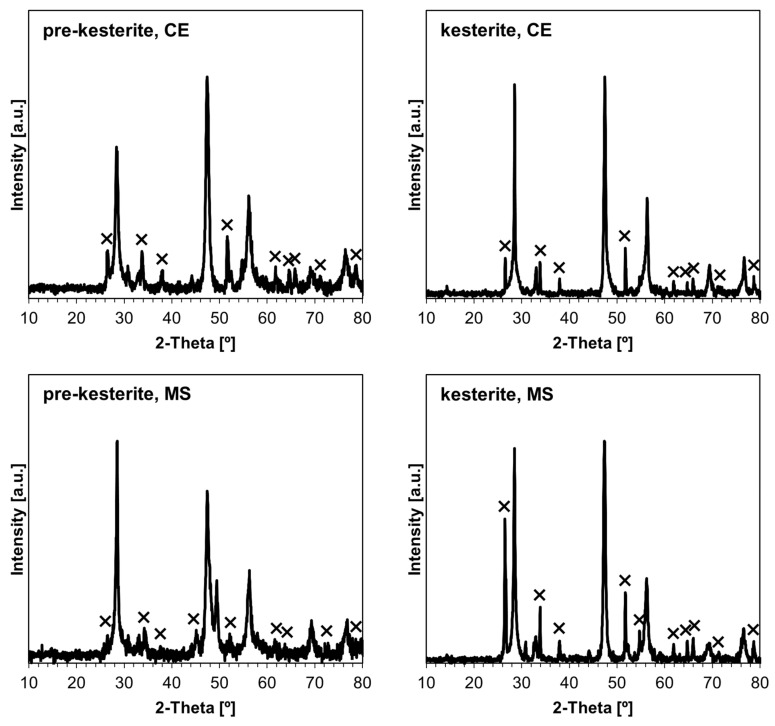
XRD patterns for nanoceramics prepared via the HP-HT sintering of nanopowders in the CE system (**top** row) and MS system (**bottom** row). Symbols (✖) are in the peak positions for cassiterite SnO_2_, whereas the unmarked peaks are for the major cubic phase in the pellets.

Figure 9 shows the XRD patterns for the sintered pellets of Cu_2_ZnSnS_4_ in both systems, and Table 3 includes the calculated structure parameters, average crystallite sizes, and phase contents. It is apparent that the compound “survives” the extreme pressure and high-temperature sintering conditions, and there are no clear signs of its decomposition. Yet, somewhat unexpectedly, a phase transition of the tetragonal kesterite to the cubic phase characteristic for prekesterite is observed in the two pellets originally made from the kesterite powders (Figure 9; right in both rows). It is instructive to recall that the nanopowders of metastable cubic prekesterite are converted to stable tetragonal kesterite by thermal treatment at 500 °C for a few hours under a neutral gas atmosphere. It is apparent that a 3 min application of high pressure (7.7 GPa) at this temperature converts the tetragonal phase back to the cubic zincblende-type phase, with slightly larger *a*-parameters than those found in the related sintered prekesterite powders. There are no available reports on the phase changes of kesterite upon the simultaneous application of increased pressures and high temperatures. However, a recent study on the impact of pressures up to 27 GPa (though at ambient temperature conditions) on the ordered and disordered tetragonal kesterite provides evidence for phase transitions to a GeSb-type structure at ca. 15 GPa, which relaxes to a disordered cubic zincblende-type phase upon decompression [39]. Another study on phase transitions in kesterite at ambient pressure and increased temperatures supports the transition of tetragonal kesterite (I42m) to a zincblende polytype (F43m) at 870 °C [40]. In both cases, the tetragonal polytype is converted to the cubic one, which is consistent with our observations. This kesterite phase change phenomenon is accompanied by some recrystallization/crystal growth that is more pronounced in the CE system, which is also supported by a comparison between the respective average crystallite sizes in the nanopowders and the pellets (cf. Table 1 and Table 3). Additionally, for all pellets, the sintering process results in some preferential crystal growth (texture effect) that is manifested by an increased relative intensity of the (220) diffraction peak at 2-Theta ca. 47° (cf. Figure 2 and Figure 9). Another striking feature of all patterns is the presence (in addition to cubic Cu_2_ZnSnS_4_) of some tin (IV) oxide SnO_2_ (cassiterite, JCPDS 14-1445). No other likely O-bearing phases, such as copper and zinc oxides or metal sulfates, are found by XRD. This is consistent with a fairly efficient contaminant oxygen redistribution under the applied HP-HT sintering conditions and the formation of one stable crystalline oxide, SnO_2_. But this also implies some tin deficiency vs. stoichiometry for Cu_2_ZnSnS_4_ in the resulting cubic phase. Therefore, the latter appears to be rather compositionally defective in structure. 

**Figure 10:**
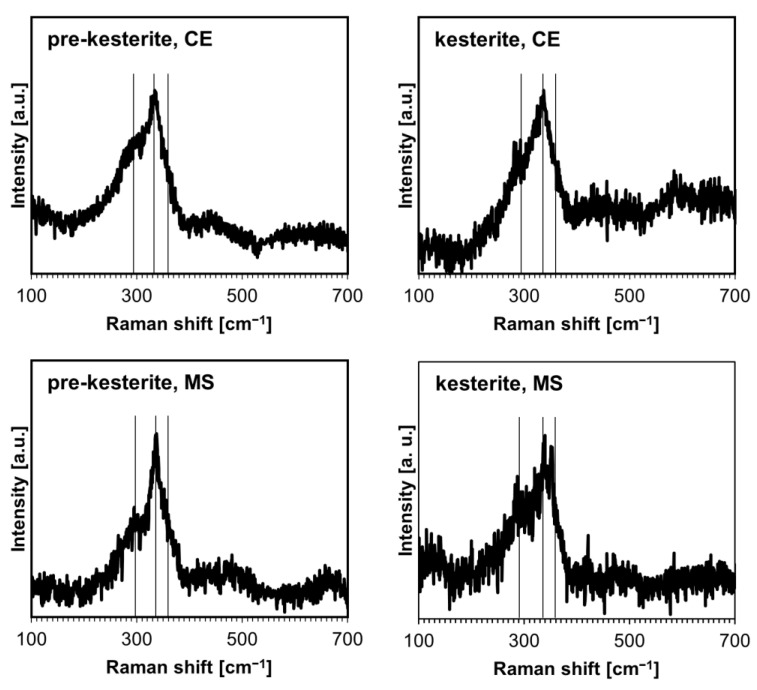
Raman spectra for sintered pellets from prekesterite and kesterite in the CE system (**top** row) and MS system (**bottom** row). Vertical lines indicate the approximate positions of the characteristic Raman bands.

The presence of the defected cubic phase in the pellets that is typical for prekesterite is further corroborated by the outcome of the ^65^Cu and ^119^Sn solid-state MAS NMR measurements for the nanoceramics. In this regard, similarly to the case of the prekesterite powders, no spectra were produced, which we ascribe to local magnetism in the structure due to d_0_ magnetism, likely linked to the presence of paramagnetic Cu^+2^ centers [20]. Furthermore, no semiconductor-specific UV-vis spectra could be collected for the pellets, as was previously observed for the non-semiconductor prekesterite powders. At the same time, the kesterite-related nature of the pellets is confirmed by Raman spectroscopy (Figure 10). The three broad bands indicated with the solid lines are typical for cubic prekesterite (cf. Figure 3 and Figure 10) [8]. Additionally, the spectra support significant disorder in the cubic phase, as indicated by the overlapping, broad Raman features, including the sintered pellets from both kesterite nanopowders.

The results of direct O and H content determinations in the pellets are shown in Table 4, and they can be related to the data in Table 2 for the parent nanopowders. In the CE system, the initial powder of kesterite and the resultant sintered pellet are characterized by similar O and H contents, whereas for the prekesterite case, slightly lesser contents are recorded. Of note, the respective lower H content, i.e., 0.13% (Table 4) vs. 0.37% (Table 2) is indicative of higher amounts of adsorbed H_2_O in the powder. After adjusting for this effect, the no-water O contents in the pellet and in the initial powder are similar. In the MS system, in both of the cases of the sintered pellets, the O and H contents are clearly higher than in the parent powders. Again, the adsorbed H_2_O is substantially responsible for this effect, as supported by the increased levels of the H contents, i.e., 0.44% vs. 0.12% (prekesterite) and 0.30% vs. 0.07% (kesterite). 

Despite the considerable attention paid to the strict sample handling and standardized analytical procedures, they are somewhat scattered with regard to sample exposure time to air. Finally, the data are consistent with the significant propensity of the kesterite materials (both nanopowders and sintered pellets) to partial oxidation when exposed to humid air, possibly for periods of time from minutes to a few hours. This is compounded in the relatively high surface area of the nanopowders by enhanced adsorption of O-bearing gases, e.g., H_2_O, O_2_, and CO_2_, that can promote further sample oxidation at the high temperature of sintering.

## 3. Materials and Methods

### 3.1. Preparation of Kesterite Nanopowders

Two precursor systems were used to make the starting powders via high-energy ball milling (Pulverisette 7 model, Fritsch, Idar-Oberstein, Germany), as already described by us in earlier reports. The first system [8], labeled as CE, was made of the constituent elements in stoichiometric proportions, i.e., copper Cu, zinc Zn, tin Sn, and sulfur S, with 2 at% excess of S, which were “wet”-milled in xylene for 16 h, 1000 rpm. Upon xylene evaporation, the resulting blackish solid prekesterite was characterized and, subsequently, used in the thermal treatment at 500 °C under argon for 6 h, yielding black kesterite nanopowders. In the second system [20], labeled as MS, a stoichiometric mixture of the metal sulfides, i.e., copper (I) sulfide Cu_2_S, zinc (II) sulfide ZnS, tin (II) sulfide SnS, and sulfur S with 2 at% excess of S, was “wet”-milled in xylene for 20 h, 900 rpm. After drying (as previously), the prekesterite nanopowder was sampled for characterization and then used in the thermal treatment at 500 °C under argon for 6 h, to yield the black powder of kesterite.

### 3.2. High-Pressure and High-Temperature Sintering

In both systems, after removing xylene by evaporation, all samples for sintering were sealed in glass vials under a vacuum. Upon vial opening, the powders were removed and briefly handled in the air immediately prior to HP-HT processing. Specifically, around 0.2 g of each powder was sintered in a high-pressure toroid cell at 500 °C, 7.7 GPa, 3 min, yielding a black ceramic pellet: D = 4 mm; thickness ca. 2–3 mm (Figure 8, insert). For characterization, the pellets were stored in a desiccator.

### 3.3. Sample Labeling

The materials/samples were labeled to show their original precursor system, the stage of the thermal processing of the powders (raw powder/prekesterite or annealed at 500 °C powder/kesterite), and the final sintering stage. The CE symbol was used for the system that was made of the constituent elements, and MS was used for the system made of the metal sulfides.

### 3.4. Characterization

SEM data were recorded with a Hitachi Model S-4700 scanning electron microscope (Hitachi, Tokyo, Japan). Powder XRD determinations were carried out for all nanopowders and sintered nanoceramics on Empyrean PANalytical (Malvern, UK), Cu Kα source, 2Θ = 10–110°, and the average crystallite sizes were estimated from Scherrer’s equation, applying the Rietveld refinement method. Raman spectroscopy was carried out on a WITec Alpha 300M+ spectrometer (WITec, Ulm, Germany) equipped with Zeiss optics (50×) and a 488 nm diode laser. Four accumulations of 30 s scans were collected at each point. Baseline subtraction was accomplished with WITec’s software (ProjectFive Plus, WITec, Ulm, Germany). Deconvolution of spectra was carried out using a mixed Gaussian–Lorentzian curve fitting. FT-IR spectroscopy (Nicolet 380, Thermo Electron Corp., Waltham, MA, USA) was carried out for KBr pellets containing about 1 mg of sample. Solid-state MAS NMR spectra were recorded on the APOLLO console (Tecmag) at a magnetic field of 7.05 T with the Bruker HP-WB high-speed MAS probe equipped with the 4 mm zirconia rotor and a KEL-F cap, which was used to spin the sample. The ^65^Cu NMR spectra were determined at 85.11 MHz, with a spinning speed of 4 kHz. The frequency scale in ppm was in reference to the ^65^Cu resonance of CuCl. The ^119^Sn NMR spectra were measured at 111.68 MHz, with a spinning speed of 4 kHz. The frequency scale in ppm was secondary-referenced to the central transition of the SnS spectrum located at −299 ppm. UV-vis measurements were accomplished with a Perkin-Elmer spectrophotometer Lambda 35 equipped with a 50 mm integrating sphere for the powder samples. TG-DTA/MS data were collected with the Netzsch STA449 F3 Jupiter thermal analyzer coupled with the QMS 403C Aëolos mass spectroscope for the analysis of the evolving gases recorded from room temperature to 1000 °C. The oxygen and hydrogen contents were directly determined with an ONH836 elemental analyzer (Leco Corporation, St. Joseph, MI, USA) using typically 0.01–0.02 mg of a sample. Helium densities were obtained with a Micromeritics AccuPyc 1340 pycnometer (Norcross, GA, USA). Ten helium purges/measurements were acquired for each sample, resulting in the average helium density d_He_ and standard deviation values. The d_He_ values for all samples were rounded up to the nearest 0.01 g/cm^3^ to show them with accuracy exceeding one standard deviation in each case. BET-specific surface areas were determined from low-temperature nitrogen adsorption on Micromeritics Gemini 2380 (Norcross, Ga, USA). The Vicker’s hardness (HV) tests were performed on FutureTech FM-700 tester (Future-Tech Corp., Fujisaki, Japan) with a 300 g-force load for 10 s. Vicker’s hardness H_V_ and standard deviation SD values were calculated in GPa units as an average of 10 measurements. For the sake of discussion, the H_V_ values were rounded up to the nearest 0.1 GPa, i.e., shown with accuracy exceeding one standard deviation value in all samples.

## 4. Conclusions

Valuable insight into the nature of various semiconductor kesterite Cu_2_ZnSnS_4_ nanopowders, as well as into preparations of the novel nanoceramics via HP-HT sintering, was gained via the combination of several characterization methods, including direct oxygen and hydrogen determinations. More specifically, the data on the often-overlooked oxygen aspect in kesterite synthesis and manipulations pose nontrivial questions related to the semiconductor’s chemical stability. It is clear that the investigated kesterite material forms are visibly affected by exposure to ambient air. The H_2_O-assisted oxidation of particle surfaces proceeds mostly toward the formation of metal sulfates. Interestingly, under HP-HT sintering conditions, a major oxidation crystalline product appears to be SnO_2_, which, in turn, defines the resultant non-stoichiometry of the compound. Although such gradual changes initially take place on particle surfaces, they will impact the compound’s bulk properties with time. From yet another perspective, the room-temperature-stable tetragonal kesterite nanopowders are shown to be structurally unstable under HP-HT conditions and are converted to a cubic zincblende-type prekesterite phase in the nanoceramics upon their decompression to ambient conditions. Such nanoceramics are defunct of the semiconductor properties that are otherwise characteristic for tetragonal kesterite. They can still be used, for instance, as a sputtering target to make thin layers of the compound to be later converted to the PV-active tetragonal polytype. Additionally, to maintain its reproducible properties, it is imperative to limit kesterite exposure to the air during synthesis, storage, and processing and, accordingly, normalize all the steps in the manipulations and analysis.

## Figures and Tables

**Table 1:** Lattice constants, *a* and *c*, and the average crystallite sizes, *D_av_*, for cubic rekesterite and tetragonal 500 °C-annealed kesterite nanopowders prepared from two precursor systems.

	Cubic Zincblende-Type Prekesterite		Disordered Tetragonal Kesterite	
	a [Å]	D_av_ [nm]	a, c [Å]	D_av_ [nm]
CE system {2Cu + Zn + Sn + 4S}	a = 5.49	8	a = 5.44 c = 10.77	14
MS system {Cu_2_S + ZnS + SnS + S}	a = 5.54	10	a = 5.43 c = 10.78	16

**Table 2:** The oxygen (O) and hydrogen (H) contents directly determined in prekesterite and kesterite nanopowders prepared in the CE and MS precursor systems. Standard deviations (SD) of the determinations are shown in parentheses.

	Powders in CE System		Powders in MS System	
	Prekesterite	Kesterite	Prekesterite	Kesterite
O-content (SD) [wt%]	5.06 (0.005)	1.65 (0.01)	3.85 (0.26)	5.36 (0.22)
H-content (SD) [wt%]	0.37 (0.01)	0.04 (0.002)	0.12 (0.01)	0.07 (0.01)

**Table 3:** Lattice constants *a,* average crystallite sizes *D_av_*, and the contents of the cubic phase present in the sintered pellets prepared from the nanopowders in the two precursor systems. Note that, in each case, cassiterite SnO_2_ phase content adds up to 100% with cubic phase content.

	Sintered Prekesterite Powders			Sintered Kesterite Powders		
	a [Å]	D_av_ [nm]	Cubic Phase Content [%]	a [Å]	D_av_ [nm]	Cubic Phase Content [%]
CE system	5.41	11	89	5.42	46	74
MS system	5.41	20	96	5.43	21	85

**Table 4:** The oxygen (O) and hydrogen (H) contents directly determined in the sintered pellets that were made from the prekesterite and kesterite nanopowders in the CE and MS precursor systems. Standard deviations (SD) of the determinations are shown in parentheses.

	Pellets in CE System Prepared From Nanopowders of		Pellets in MS System Prepared From Nanopowders of	
	Prekesterite	Kesterite	Prekesterite	Kesterite
O-content (SD) [wt%]	3.94 (0.44)	1.75 (0.04)	6.75 (0.08)	9.25 (0.14)
H-content (SD) [wt%]	0.13 (0.03)	0.02 (0.01)	0.44 (0.01)	0.30 (0.004)

## Data Availability

The data presented in this study are available on request from the corresponding author.
